# Chloroplast genome sequence confirms distinctness of Australian and Asian wild rice

**DOI:** 10.1002/ece3.66

**Published:** 2012-01

**Authors:** Daniel L E Waters, Catherine J Nock, Ryuji Ishikawa, Nicole Rice, Robert J Henry

**Affiliations:** 1 Southern Cross Plant Science, Southern Cross UniversityLismore, Australia; 2 Faculty of Agriculture and Life Science, Hirosaki UniversityHirosaki Aomori, Japan; 3 Queensland Alliance for Agriculture and Food Innovation, University of QueenslandBrisbane, Australia

**Keywords:** Chloroplast genome, *Oryza meridionalis*, *Oryza rufipogon*, *Oryza sativa*, wild rice

## Abstract

Cultivated rice (*Oryza sativa*) is an AA genome *Oryza* species that was most likely domesticated from wild populations of *O. rufipogon* in Asia. *O. rufipogon* and *O. meridionalis* are the only AA genome species found within Australia and occur as widespread populations across northern Australia. The chloroplast genome sequence of *O. rufipogon* from Asia and Australia and *O. meridionalis* and *O. australiensis* (an Australian member of the genus very distant from *O. sativa*) was obtained by massively parallel sequencing and compared with the chloroplast genome sequence of domesticated *O. sativa*. *Oryza australiensis* differed in more than 850 sites single nucleotide polymorphism or indel from each of the other samples. The other wild rice species had only around 100 differences relative to cultivated rice. The chloroplast genomes of Australian *O. rufipogon* and *O. meridionalis* were closely related with only 32 differences. The Asian *O. rufipogon* chloroplast genome (with only 68 differences) was closer to *O. sativa* than the Australian taxa (both with more than 100 differences). The chloroplast sequences emphasize the genetic distinctness of the Australian populations and their potential as a source of novel rice germplasm. The Australian *O. rufipogon* may be a perennial form of *O. meridionalis*.

## Introduction

*Oryza sativa*, the dominant cultivated rice species, shares the genus *Oryza* with approximately 22 other species (Khush 1997). Genetically, the primary factor that differentiates the species within this genus is genome organization and ploidy as determined by hybrid chromosome pairing behavior (Lu et al. 1997). On the basis of this evidence, six diploid genomes, AA, BB, CC, EE, FF, and GG, and four allotetraploid genomes BBCC, CCDD, HHKK, and HHJJ have been identified (Lu et al. 2009). *Oryza sativa* belongs to the AA genome group along with the only other cultivated species *O. glaberrima,* and the wild rice species *O. rufipogon, O. nivara, O. longistaminata*, *O. glumaepatula*, *O. barthii,* and *O. meridonalis* (Ge et al. 1999).

Within *O. sativa* there are two subspecies, *indica* and *japonica*. These subspecies are the product of either one (Molina et al. 2011) or two independent domestication events (Sweeney and McCouch 2007). Regardless of whether there have been one or two independent domestication events, the process of domestication and more recently selection during plant breeding has resulted in cultivated *O. sativa* resting on a relatively narrow genetic base. Because of this, AA genome wild rice species have been a valuable source of new genes and alleles for resistance to a range of pests and diseases (Brar and Khush 1997). However, Asian wild rice is in close contact with cultivated rice and there is constant gene flow between the cultivated and wild populations that contaminates the Asian wild rice gene pool with cultivated alleles, an example of which is the shattering gene being found in wild rice (Sweeney and McCouch 2007). In contrast, with the exception of failed attempts to establish commercial rice growing in the Northern Territory and Western Australia in the 1950s, the Burdekin irrigation area in the early 1990s (Anonymous, 2005) and the more recent crop of 650 ha in Western Australia, Australian wild rice has been largely genetically isolated from cultivated rice. Because of this, the Australian wild rice gene pool has not been contaminated with cultivated *Oryza* alleles to the same extent as the Asian wild rice gene pool making Australian wild rice a potential source of valuable alleles for rice breeding.

**Figure 1:**
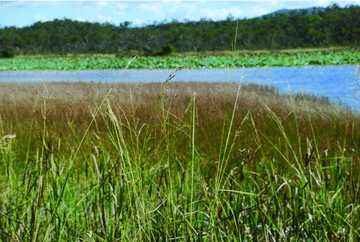
*Oryza rufipogon* growing in its native habitat near Mareeba, north Queensland, Australia. Anther length is the primary morphological feature used for discrimination between Australian *O. rufipogon* (>3–7.4 mm) and *O. meridonalis* (1.5–2.5 mm).

**Figure 2:**
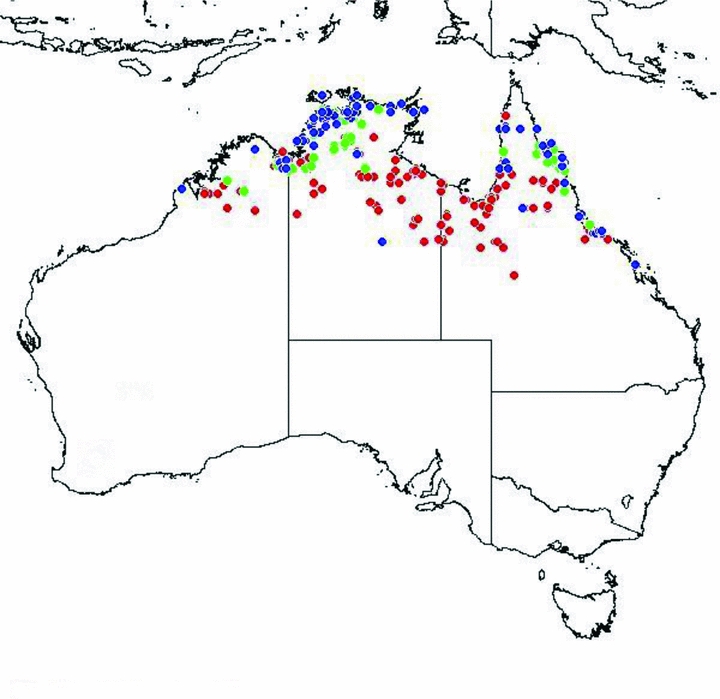
Estimated distribution of Australian *Oryza* species: *O. rufipogon* in blue, *O. meridionalis* in green, and *O. australiensis* in red. Based on occurrence records provided by Australia's Virtual Herbarium, accessed through the Atlas of Living Australia website (http://www.ala.org.au).

The AA genome *Oryza* species endemic to northern Australia are *O. meridionalis* and *O. rufipogon* (Figs. 1 and 2). These species are primarily distinguished by anther size, Australian *O. rufipogon* does not share the small anthers of *O. meridionalis* and life history, *O. meridionalis* is an annual species while *O. rufipogon* is a perennial species. These species grow in close proximity to each other, *O. rufipogon* grows in transient pools and ponds where some water persists during the dry season while *O. meridionalis* grows on the periphery of these same bodies of water surviving the dry season as seed. This is analogous to the relationship between Asian *O. rufipogon* and *O. nivara. Oryza nivara* has been variously described as an annual species that grows in swamps, which dry out during the dry season, unlike *O. rufipogon* that grows in deep permanent water, or as an ecotype of *O. rufipogon* with which it shows a continuous distribution in location and morphology (Naredo et al. 1997).

Despite many different approaches, the taxonomy of the AA genome Oryza remains a work in progress. The relationship between *O. rufipogon, O. nivara,* and *O. meridionalis* is unclear. Analysis of chromosome pairing has confirmed that *O. meridionalis* and *O. rufipogon* are AA genome species (Lu et al. 1997). In common with most early molecular taxonomic treatments, however, *O. rufipogon* samples used by Lu et al. (1997) were sourced from Asia only and therefore did not provide evidence of the relationship between Australian *O. rufipogon* and *O. meridionalis.* Experimental crosses between Australian *O. rufipogon* and *O. meridionalis* produced interspecific hybrids although fertility and seed set of the hybrids was low (Naredo et al. 1997). Restriction fragment length polymorphism (RFLP) and Short interspersed elements (SINE) data derived from sample sets including both Australian and Asian *O. rufipogon* and *O. meridionalis* suggest these species are different (Wang et al. 1992; Xu et al. 2005), and that Australian *O. rufipogon* is more closely related to Asian *O. rufipogon* than it is to *O. meridionalis* (Wang et al. 1992).

Phylogenies derived from nuclear data can be problematic because recombination may confound phylogenetic resolution and lead to the construction of inconsistent trees (Poke et al. 2006; Takahashi et al. 2008). Plastid sequence data, in contrast, are haploid and offer the advantages of high copy number without recombination. As the number of informative characters increases, so does phylogenetic resolution. Next Generation (or massively parallel) sequencing can cost-effectively sample large numbers of informative characters and hence dramatically increase phylogenetic resolution. Whole chloroplast genome sequencing for phylogenetic analysis without prior isolation or amplification is now relatively straightforward for plant species (Nock et al. 2011). This approach captures a large quantity of chloroplast sequence data, and whole plastome sequences can be used to resolve phylogenetic relationships among even closely related species (e.g., Parks et al. 2009; Zhang et al. 2011). We have applied this approach to the analysis of the relationship between Australian and Asian wild AA genome wild rice populations and found the Australian wild rice species to be genetically distinct from closely related Asian AA genome wild rice.

## Material and Methods

### Plant materials

The Asian *O. rufipogon* strain was collected from GPS location N9 59.376 E105 39.883, Can Tho, Vietnam (Australian Plant DNA Bank Number–AC11–1008369). Australian *O. rufipogon* was sourced from the Australian Tropical Crops and Forages Collection, Biloela (Ref Number-AusTRCF 309313; Australian Plant DNA Bank Number–AC01–1002323; originally collected 23 March, 1994, 0.9K west Gilbert River Bridge in Gulf Development Rd, latitude = –18.206, longitude = 142.865). *Oryza meridionalis* was sourced from the Australian Tropical Crops and Forages Collection, Biloela (Ref Number-AusTRCF 300118_B; originally collected Northern Territory, Australia). Anther length was the primary morphological feature used for discrimination between Australian *O. rufipogon* (>3–7.4 mm length) and *O. meridonalis* (1.5–2.5 mm length).

### DNA extraction and sequence analysis

DNA was extracted from leaf tissue of four individuals plants from each accession using a Qiagen DNeasy Plant kit (Qiagen, Hilden, Germany). Approximately 3 µg of total DNA from each sample was prepared for sequencing according to Illumina genomic, paired-end sample preparation protocol (Part # 1005063 Rev. A). DNA was sheared using an adaptive focused acoustics method on a Covaris S2 device with the following settings: duty cycle 10%; intensity 5; cycles per burst 200 for 180 sec at 6°C.

Ligation products were purified by agarose gel electrophoresis (2% agarose, 120 V for 120 min). Fragments of predominantly 500 base pairs (bp) were excised from the gel and the products isolated with a QIAquick Gel Extraction kit (Qiagen, Hilden, Germany)without heating. PCR products were further purified with a QIAquick PCR Purification kit (Qiagen, Hilden, Germany) and quantified using a DNA 1000 chip on an Agilent BioAnalyzer 2100 (Agilent Technologies, Santa Clara, CA, USA). Approximately 4 pmol per individual and 3 pmol of the PhiX control lane were sequenced for 76 × 2 cycles on an Illumina Genome Analyser (GAIIx) (Illumina, San Diego, CA, USA.). Base calling was performed with Illumina software Pipeline 1.5.

Paired-end sequence reads were trimmed of low quality data with a quality score limit of 0.01, and adaptor sequence in CLC Genomics Workbench 4.0.3 (http://www.clcbio.com). Reads of less than 30 bp in length were discarded. Trimmed short read sequences were assembled by read mapping to a cultivated rice (*O. sativa* spp. *japonica* var. Nipponbare) chloroplast genome reference sequence (Genbank accession GU592207). Read mapping was undertaken in CLC Genomics Workbench with the following long-read parameters: global alignment, length fraction 0.9, similarity index 0.9, mismatch cost 3, deletion, and insertion costs 3. Match mode was random to allow for assembly of both inverted repeat regions and repetitive elements. In order to avoid contribution of less abundant nuclear and mitochondrial reads to the final consensus sequence, conflict resolution mode was vote majority.

Consensus sequences for *O. rufipogon* and *O. meridionalis* were exported to Geneious 5.3 (http://www.geneious.com) and aligned with chloroplast genome sequences from Genbank (Fig. 1) using Mauve (Darling et al., 2004). Genbank accessions included in the alignment were *O. sativa japonica* GU592207, *O. nivara* AP006728, *O. sativa indica* AY522329, and *O. australiensis* GU592209.

Appropriate nucleotide substitution models were selected using Modeltest and MrModeltest (Posada and Crandall 1998). Aligned data were analyzed under maximum parsimony (MP) and maximum likelihood (ML) criteria using the TVM + I model (G = 0.92) in PAUP* (http://www.paup.csit.fsu.edu) with gaps were treated as missing data. Heuristic searches were conducted with 200 random addition replicates and tree bisection-reconnection (TBR) branch swapping. *Oryza australiensis* was the outgroup in rooted trees with 2000 bootstrap replicates to evaluate nodal support. Bayesian phylogenetic analysis was conducted using MrBayes 3.1 (Ronquist and Huelsenbeck, 2004) using the GTR + I model. Two independent runs of 1 × 10^6^ Monte Carlo Markov Chains (MCMC) were performed following burn in of 1 × 10^5^ MCMC, each starting with a different random tree. Nodal support for Bayesian consensus trees was evaluated by posterior probability distribution. Consensus sequences were annotated using Dual Organellar Genome Annotator (DOGMA) (Wyman et al. 2004) and manually adjusted as needed before submission to Genbank.

## Results

After trimming, 96.6%, 97.78%, and 96.43% of paired-end reads with average lengths of 72.1 bp, 71.5 bp, and 71.7 bp were retained for *O. rufipogon* (Aust), *O. rufipogon* (Asian), and *O. meridionalis,* respectively (Table 1). Consensus sequence lengths generated from reference mapping of Illumina sequence reads were 134,558 base pairs (bp) for *O. meridionalis*, 134,544 bp for *O. rufipogon* (Asian), and 134,557 bp for *O. rufipogon* (Aust).

**Table 1:** Summary statistics for wild rice chloroplast genome assembly. Sequence reads were mapped to a cultivated rice *O. sativa* spp. *Japonica* var. Nipponbare reference (Genbank accession GU592207)

Species	Source	Paired-end reads	Average read length	Reads aligning to chloroplast genome (%)	Median coverage	Consensus sequence (bp)	Accession number
*O. rufipogon*	Australia	67,046,275	72.0	12.98	4870	134,557	JN005833
*O. rufipogon*	Vietnam	62,321,354	71.5	2.27	781	134,544	JN005832
*O. meridionalis*	Australia	46,409,181	71.7	4.81	1231	134,558	JN005831

**Figure 3:**
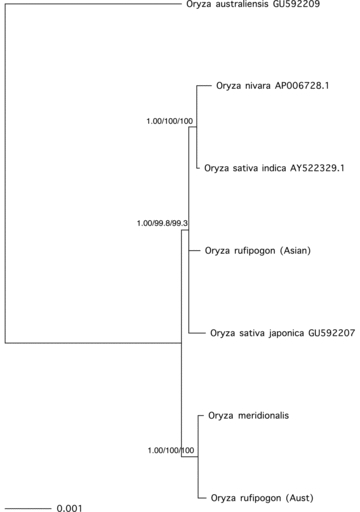
Bayesian phylogenetic tree illustrating the relationships among *Oryza* chloroplast genome sequences. The same topology was obtained by Bayesian analysis, maximum likelihood (ML), and maximum parsimony (MP). Nodal support is shown above branches as Bayesian (posterior probability)/ML/MP (percent bootstrap). Scale is substitutions per site. *Oryza australiensis* is the outgroup. Genbank accession numbers follow previously published sequences.

The alignment of seven chloroplast genomes was 134,701 bp in length. One of the inverted repeats (IR) was excluded from the alignment prior to phylogenetic analysis. The modified alignment was 113,897 bp in length. Of the 978 variable characters, 90 were parsimony informative. Optimal phylogenetic trees obtained by MP (968 steps; consistency index = 1.00), ML (-lnL = 162,141.42), and Bayesian analysis were concordant. The relationship between *O. sativa japonica* and *O. rufipogon* (Asia) was unresolved. There was strong support (MP, ML bootstrap ≥ 99.3%; Bayesian posterior probability = 1.00) for an Australian A genome clade containing *O. rufipogon* (Aust) and *O. meridionalis*, and for an Asian A genome clade containing *O. rufipogon* (Asia), *O. nivara,* and Asian cultivated rice *O. sativa* ssp. *japonica and O. sativa* ssp. *indica* (Fig. 3). *O. rufipogon* and *O. meridionalis* chloroplast genomes from Australia differed by only 32 positions. The monophyly of Australian A genome wild rice was supported by 38 shared derived characters or synapomorphic SNPs (Table 2). Homoplasy in the dataset was not detected (homoplasy index = 0.00) and there were no derived characters shared between *O. rufipogon* chloroplast genome sequences from Asia and Australia. The monophyly of wild and cultivated Asian rice was supported by 16 synapomorphic SNPs.

**Table 2:** Synapomorphic SNPs (in bold italics) for Australian and Asian AA genome clades of wild and cultivated rice included in this study. Sequence position is according to the reference sequence, *O. sativa* spp. *japonica* var. Nipponbare (GU592207)

Position	Asian Clade	Australian Clade	Outgroup	Gene
**LSC**				
448	A	***G***	A	psbA
817	A	***G***	G	psbA
1522	G	***A***	G	*
2221	***T***	G	G	matK
3068	***T***	*G*	A	matK
3578	***A***	T	T	*
4547	K	***A***	T	*
6247	C	***T***	C	*
6673	A	***G***	A	*
8144	C	***T***	C	*
11735	C	***T***	C	*
11758	C	***T***	C	*
13768	***C***	A	A	*
13983	A	***C***	A	*
15330	***C***	G	G	*
15634	***C***	G	G	*
16060	A	***C***	A	*
16317	T	***C***	T	*
16842	T	***A***	T	*
17267	***G***	***A***	T	*
17351	T	***C***	T	*
18173	C	***G***	C	*
18863	A	***C***	A	*
22455	A	***C***	A	rpoC1
24758	***G***	T	T	*
25016	A	***G***	A	rpoC2
27965	A	***C***	A	rpoC2
29901	C	***T***	C	rps2
31858	A	***G***	A	*
32596	C	***T***	C	*
33680	G	***A***	G	atpF
41834	C	A	A	*
43643	***A***	G	G	*
50078	***T***	C	C	*
50146	***T***	C	C	*
55344	A	***G***	A	rbcL
60191	C	***T***	C	*
61211	G	***T***	G	*
61382	G	***T***	G	psbJ
63315	***T***	A	A	*
64817	T	***A***	T	*
65577	***T***	C	C	*
IR_A_				
90581	T	*G*	T	*
**SSC**				
104189	T	***G***	T	*
105076	G	***A***	G	*
105922	A	***C***	A	*
106562	T	***G***	T	ndhD
106705	T	***G***	T	ndhD
108465	***T***	**G**	G	*
110607	*G*	A	A	*
110844	A	***G***	A	*
**IR_B_**				
124575	A	***C***	A	*

* Intergenic region.

LSC = large single copy; SSC = small single copy; IR = inverted repeats.

## Discussion

Previous analyses suggest the perennial Australian wild rice *O. rufipogon* is more closely related to Asian *O. rufipogon* than it is to the annual Australian wild rice *O. meridionalis* (Wang et al. 1992; Xu et al. 2005). Here we show that the plastome of Australian *O. rufipogon* is more closely related to *O. meridionalis* than it is to Asian *O. rufipogon,* demonstrating Australian AA genome wild rices are distinct from Asian wild and cultivated rice. In addition to this, SNP haplotyping of all individuals of *O. meridionalis* so far examined sourced from both Australia directly and accessions held by the National Bio-resources project (NBRP) of Japan carry the *O. meridionalis* type of chloroplast DNA reported here (data not shown).

This study utilized whole chloroplast sequence data that brings particular advantages to the analysis. Plastid genomes do not undergo recombination and are present in high copy number relative to nuclear loci (Takahashi et al. 2008). This attribute has been exploited for many studies including plant barcoding (CBOL 2009). However, until recently, a relatively small number of nucleotides have been routinely sampled for chloroplast based plant identification. For example, approximately 1450 base pairs from *rbcL* and *matK* were used as the foundation for a DNA barcode for land plants. Although useful, this approach only allowed discrimination of 72% species in a sample set of 907 species. The complete chloroplast genome has two orders of magnitude more information than the conventional *rbcL* and *matK* plant barcode loci and by accessing a greater number of characters, greater phylogenetic resolving power is possible.

Chloroplast DNA is maternally inherited in most angiosperms (Hagemann 2010). Interspecific hybridization can lead to “chloroplast capture” whereby the plastome of one species introgresses into another, and this has been used to explain inconsistencies between chloroplast and nuclear gene trees. Historical or more recent hybridization between sympatric populations of *O. meridionalis* and *O. rufipogon* in Australia provides an alternative explanation for the observed results.

During domestication, Asian cultivated rice went through a significant bottleneck and brought with it only 10–20% of the genetic diversity found within its progenitor species, *O. rufipogon* (Kovach and McCouch 2008). The genetic diversity within wild rice has been exploited to enhance cultivated rice, primarily by improving yield and agronomic traits (Kovach and McCouch 2008). In order to most effectively exploit the genetic diversity within wild rice, the hybrid offspring needs to be fertile. *Oryza sativa* is an AA genome species and other AA genome wild species are the most accessible in terms of generating fertile hybrid offspring, including *O. rufipogon*, *O. nivara*, *O. barthii*, *O. longistaminata*, *O. glumaepatula,* and *O. meridionalis*. Crosses between *O. meridionalis,* Australian *O. rufipogon,* and other AA genome *Oryza* species generate fertile hybrids and so the alleles within these species are available to *O. sativa* breeding programs following conventional crossing regimes (Naredo et al. 1997). Because Australian AA genome wild rice has been largely isolated from *O. sativa* during the course of *O. sativa* domestication and cultivation, the Australian wild rice is a valuable source of novel alleles for rice improvement.

*Oryza nivara* is variously described as an annual ecotype of Asian *O. rufipogon* or as a separate species (Zheng and Ge 2010). The relationship between Australian *O. rufipogon* and *O. meridonalis* is somewhat similar with *O. meridonalis* until recently being described as an annual form of Australian *O. rufipogon* (Wang et al. 1992). In both cases the key differentiating feature is the life history of these species or ecotypes. Our results suggest the divergence of the Australian and Asian AA genome rice predates the divergence of *O. nivara* from Asian *O. rufipogon* and Australian *O. rufipogon* from *O. meridonalis*. If so, the appearance of the annual and perennial habits in each of these species and or ecotypes in Australia and Asia were separate events. Genetic and genomic analysis of Asian and Australian *O. rufipogon, O. nivara,* and *O. meridonalis* may allow identification of loci or gene networks that differentiate between the perennial and annual species or ecotypes in each of these cases.

## References

[b1] Anonymous (2005). The biology and ecology of rice (Orzya sativa L.) in Australia.

[b2] Brar DS, Khush GS (1997). Alien introgression in rice. Plant Mol. Biol.

[b3] Consortium for the Barcode of Life (CBOL) Plant Working Group (2009). A DNA barcode for land plants. Proc. Natl. Acad. Sci. USA.

[b4] Darling AC, Mau B, Blattner FR, Perna NT (2004). Mauve: multiple alignment of conserved genomic sequence with rearrangements. Genome Res.

[b5] Ge S, Sang T, Lu B-R, Hong D-Y (1999). Phylogeny of rice genomes with emphasis on origins of allotetraploid species. Proc. Natl. Acad. Sci. USA.

[b6] Hagemann R (2010). The foundation of extranuclear inheritance: plastid and mitochondrial genetics. Mol. Genet. Genomics.

[b7] Khush GS (1997). Origin, dispersal, cultivation and variation of rice. Plant Mol. Biol.

[b8] Kovach MJ, McCouch SR (2008). Leveraging natural diversity: back through the bottleneck. Curr. Opin. Plant Biol.

[b9] Lu B, Naredo EB, Juliano AB, Jackson MT (1997). Hybridization of AA genome rice species from Asia and Australia II. Meiotic analysis of *Oryza meridionalis* and its hybrids. Genet. Res. Crop Evol.

[b10] Lu F, Jetty JSS, Sanyal A, Zhang S, Song R, Chen J, Li G, Sui Y, Song X, Chen Z, et al (2009). Comparative sequence analysis of MONOCULM1-orthologous regions in 14 *Oryza* genomes. Proc. Natl. Acad. Sci. USA.

[b11] Molina J, Sikora M, Garud N, Flowers JM, Rubinstein S, Reynolds A, Huang P, Jackson S, Schaal BA, Bustamante CD, et al (2011). Molecular evidence for a single evolutionary origin of domesticated rice. Proc. Natl. Acad. Sci. USA.

[b12] Naredo EB, Juliano AB, Lu B-R, Jackson MT (1997). Hybridization of AA genome rice species from Asia and Australia I. Crosses and development of hybrids. Genet. Res. Crop Evol.

[b13] Nock CJ, Waters DLE, Edwards MA, Bowen SG, Rice N, Cordeiro GM, Henry RJ (2011). Chloroplast genome sequences from total DNA for plant identification. Plant Biotechnol. J.

[b14] Parks M, Cronn R, Liston A (2009). Increasing phylogenetic resolution at low taxonomic levels using massively parallel sequencing of chloroplast genomes. BMC Biology.

[b15] Poke FS, Martin DP, Steane DA, Vaillancourt RE, Reid JB (2006). The impact of intragenic recombination on phylogenetic reconstruction at the sectional level in Eucalyptus when using a single copy nuclear gene (cinnamoyl CoA reductase). Mol. Phylogenet. Evol.

[b16] Sweeney M, McCouch SR (2007). The complex history of the domestication of rice. Ann. Bot.-London.

[b17] Takahashi H, Sato Y-I, Nakamura I (2008). Evolutionary analysis of two plastic DNA sequences in cultivated and wild species of *Oryza*. Breed. Sci.

[b18] Wang ZY, Second G, Tanksley SD (1992). Polymorphism and phylogenetic relationships among species in the genus *Oryza* as determined by analysis of nuclear RFLPs. Theor. Appl. Genet.

[b19] Wyman SK, Jansen RK, Boore JL (2004). Automatic annotation of organellar genomes with DOGMA. Bioinformatics.

[b20] Xu JH, Kurata N, Akimoto M, Ohtsubo H, Ohtsubo E (2005). Identification and characterization of Australian wild rice strains of Oryza meridionalis and Oryza rufipogon by SINE insertion polymorphism. Genes Genet. Syst.

[b21] Zhang Y-J, Ma P-F, Li D-Z (2011). High-throughput sequencing of six bamboo chloroplast genomes: phylogenetic implications for temperate woody bamboos (Poaceae: Bambusoideae). PLoS One.

[b22] Zheng X-M, Ge S (2010). Ecological divergence in the presence of gene flow in two closely related Oryza species (*Oryza rufipogon* and *O. nivara*. Mol. Ecol.

